# Transcriptome profiling of digital flexor tendons after injury in a chicken model

**DOI:** 10.1042/BSR20191547

**Published:** 2020-06-05

**Authors:** Wei Feng Mao, Yin Xian Yu, Chen Chen, Ya Fang Wu

**Affiliations:** 1 Department of Hand Surgery, Affiliated Hospital of Nantong University, Nantong, Jiangsu, China; 2 Department of Anatomy, Medical School, Nantong University, Nantong, Jiangsu, China; 3 Department of Orthropaedic Surgery, Shanghai General Hospital, Shanghai Jiao Tong University, Shanghai, China

**Keywords:** Gene ontology, Tendon healing, Weighted gene co-expression network analysis

## Abstract

**Background**: Modulation of tendon healing remains a challenge because of our limited understanding of the tendon repair process. Therefore, we performed the present study to provide a global perspective of the gene expression profiles of tendons after injury and identify the molecular signals driving the tendon repair process.

**Results**: The gene expression profiles of flexor digitorum profundus tendons in a chicken model were assayed on day 3, weeks 1, 2, 4, and 6 after injury using the Affymetrix microarray system. Principal component analysis (PCA) and hierarchical cluster analysis of the differentially expressed genes showed three distinct clusters corresponding to different phases of the tendon healing period. Gene ontology (GO) analysis identified regulation of cell proliferation and cell adhesion as the most enriched biological processes. Kyoto encyclopedia of genes and genomes (KEGG) pathway analysis revealed that the cytokine–cytokine receptor interaction and extracellular matrix (ECM)–receptor interaction pathways were the most impacted. Weighted gene co-expression network analysis (WGCNA) demonstrated four distinct patterns of gene expressions during tendon healing. Cell adhesion and ECM activities were mainly associated with genes with drastic increase in expression 6 weeks after injury. The protein–protein interaction (PPI) networks were constructed to identify the key signaling pathways and hub genes involved.

**Conclusions:** The comprehensive analysis of the biological functions and interactions of the genes differentially expressed during tendon healing provides a valuable resource to understand the molecular mechanisms underlying tendon healing and to predict regulatory targets for the genetic engineering of tendon repair.

Tendon healing, Adhesion, Gene Ontology, Kyoto Encyclopedia of Genes and Genomes, Weighted Gene Co-expression Network Analysis, Protein–protein Interaction

## Introduction

Tendons transmit tensile forces from muscle to bone and the proper functioning of tendon relies on its structural integrity. Following an injury, tendon tissue undergoes significant changes, including infiltration of inflammatory cells, recruitment of fibroblasts, synthesis of extracellular matrix (ECM) components, and secretion of cytokines and growth factors that actively modulate the tendon repair process [1,2]. Animal and cell culture models have been developed to study cellular and molecular events that occur following tendon injury [3–5]. These studies have highlighted specific growth factors or transcription factors critical in tendon repair or regeneration. However, the mechanisms underlying tendon healing have not been fully characterized, and the management of tendon healing remains a challenge for hand surgeons [6]. The main reason for the lack of effective treatments for tendon injury is due to our limited understanding of the tendon repair process, especially at the molecular level. Therefore, a global perspective on gene expression profiles of tendons after injury, as well as the functions and interactions of the differentially expressed genes, needs to be elucidated.

Microarray technology is a powerful tool that allows the simultaneous evaluation of the expression profiles of thousands of genes. The utility of this technology can be maximized when gene expression profiles differ greatly due to the significant alterations in biological functions and molecular pathways. Microarray-based studies can identify many key genes and provide insight into the potential transcriptional regulations underlying various biological processes [7,8]. A number of microarray studies have analyzed the response of exercise- or age-induced tendon degeneration [9–12]. To our knowledge, there have been no reports on global gene expression profiling after a definitive tendon injury. Chaudhury et al. [13] reported distinct gene expression patterns in normal human rotator cuff tendons and in tendon tears of different sizes, but their study could not demonstrate the dynamic changes of gene expression after tendon injury.

To uncover the mechanisms involved in tendon healing, we carried out a transcriptome-based microarray assay in the present study. Samples were generated from the digital flexor tendons of chickens subjected to surgical transection and repair. The samples were divided into groups and assessed at several time points (from postoperative day 3 to week 6 after injury) covering the critical tendon healing period, which allowed us to understand the dynamic changes of the transcriptome during tendon healing. Bioinformatics analysis was conducted to identify key genes associated with tendon repair and to elucidate their interactions. Understanding the changes in gene expressions and the molecular pathways that actively participate in tendon healing will provide valuable insight into the mechanisms underlying tendon repair and help identify novel therapeutic targets.

## Methods

### Animal surgery and sample collection

The present study was approved by the experimental animal care committee of Nantong University, and all the animal experiments were performed in Nantong University. Adult white Leghorn chickens were obtained from the Experimental Animal Center of Nantong University. White Leghorn chickens, weighing 1.5–2.5 kg each, were chosen as the experimental model because the tendon and pulley anatomy structure of chicken toes are similar to those of humans, and chicken model is frequently used in tendon healing studies [14–16]. All experimental procedures were carried out according to the institutional animal care guidelines of Nantong University and in accordance with the ethical standards set by the Administration Committee of Experimental Animals, Jiangsu, China. The present study adheres to the ARRIVE Guidelines of animal research (Supplementary Checklist S5).

A total of 18 chickens were used, 15 of which underwent surgery for flexor digitorum profundus tendon transection and repair according to well-established protocols [17,18]. The chickens were anesthetized with intramuscular injection of ketamine (50 mg/kg body weight). After surgery, the repaired toes were immobilized in semiflexion using a cast. The chickens were caged two in one cage and allowed to walk freely with ad libitum access to water and diet. We did not observe any adverse events in the feeding process. These chickens were randomized into five groups for assessment at different time points: postoperative day 3, week 1, week 2, week 4, and week 6, with 3 chickens in each group. The remaining three chickens did not undergo surgery and were used as the control group. The chickens were humanely sacrificed under deep anesthesia with sodium pentobarbital (200 mg/kg body weight) at the time of harvest. Three individual tendon samples were collected at each time point for microarray analysis and the other three individual tendon samples were collected for quantitative real-time PCR.

### Microarray analysis

Total RNA was extracted and purified using the mirVana™ miRNA Isolation Kit (Cat#AM1560, Ambion, Austin, TX, U.S.A.) according to the manufacturer’s instructions. The RNA Integrity Number (RIN) was calculated to verify the integrity of RNA using Agilent Bioanalyzer 2100. Total RNA was amplified, labeled, and purified using the GeneChip 3’IVT Express Kit (Cat#901229, Affymetrix, U.S.A.). Labeled cRNA was hybridized to GeneChip® Chicken Genome Array (Affymetrix, US) in accordance with the manufacturer’s instruction. Raw data were normalized by MAS 5.0 algorithm in Gene Spring Software 11.0 (Agilent technologies, U.S.A.). Microarray experiments were conducted by Shanghai Biotechnology Corporation, Shanghai, China.

### Data processing

First, we determined a cut-off for probes with flags as ‘present’ in 30% samples. Then, Pearson correlation, principal component analysis (PCA), and unsupervised hierarchical clustering were performed using the R software on the remaining data to determine intergroup correlations and identify outliers. Statistical significance of gene expression differences was analyzed using one-way ANOVA for all time points. Differentially expressed genes were defined as a >2-fold change in mean expression compared with the previous time point or the control and with a *P* value of <0.05. In addition, we used the R ‘time course’ package to identify genes of interest, in which the multivariate empirical Bayes model was employed. This model has been reported to be superior to traditional *F*-statistic for the analysis of time series data [19]. Gene ontology (GO) annotation of the differentially expressed genes was performed using David online system (https://david.ncifcrf.gov/summary.jsp), and Kyoto encyclopedia of genes and genomes (KEGG) pathway annotation was performed using the R Signaling Pathway Impact Analysis (SPIA) package [20].

### Weighted gene co-expression network analysis (WGCNA)

Probes with *P*<0.05 (one-way ANOVA as described above) and with accurate gene annotations were included. Each gene was only represented by the most significant variant probe; therefore, 662 variant probes were used for analysis. WGCNA modeling was performed using the R WGCNA package, and individual modules (sets of co-regulated genes) were generated using the cutreeHybrid function [21]. The expression profile of a given module was represented by its first principal component (known as module eigengene, ME) that could explain the most variation of the module expression levels, and an ME-to-time correlation was visualized as a heatmap chart. Genes with the highest module membership values (known as ME-based connectivity, kME) were referred to as intramodular hub genes. Functional annotation for each module was performed using David online system.

### Protein–protein interaction (PPI) network analysis

We constructed an experimentally validated PPI network using genes (kME>0.05) from the co-expression network modules in two classes. We identified all experimentally verified interaction data for their corresponding proteins and homologous proteins of human and mouse genome in the STRING database (an open-access database for the analysis of protein–protein interactions [22]). Networks were visualized in a force-directed layout in Cytoscape [23]. The igraph package was used to compute network attributes and node centrality [24]. For node centrality, betweenness centrality was used.

### Quantitative real-time PCR

Validation of the Affymetrix data was performed by real-time PCR analysis with the Mastercycler ep realplex (Eppendorf 2S, Hamburg, Germany). Total RNA was isolated from the tendon segment with the Trizol reagent according to the manufacturer’s instructions (Invitrogen) and RNA was then reverse-transcribed into cDNA using an Hiscript RT kit (Vazyme, R223-01) according to the manufacturer’s protocol. Real-time PCR reactions were performed to examine gene expression using the AceQ qPCR SYBR Green Master Mix (Vazyme, Q111-02/03) and 100 nmol/l of gene-specific forward and reverse primers. A 20 µl reaction mixture contained 1 µl of cDNA from samples, 10 µl of AceQ qPCR SYBR Green Master Mix, 0.4 µl primers (10 µM) respectively, and 8.2 µl of RNase/DNase-free water. The reaction conditions were 5 min at 95°C, 10 s at 95°C and 30 s at 60°C for 40 cycles. Relative expression level for each target gene was normalized by the Ct value of GAPDH (internal control) using a 2DDCt relative quantification method.

## Results

### Transcriptional profiles of tendons during healing

**Figure 1:**
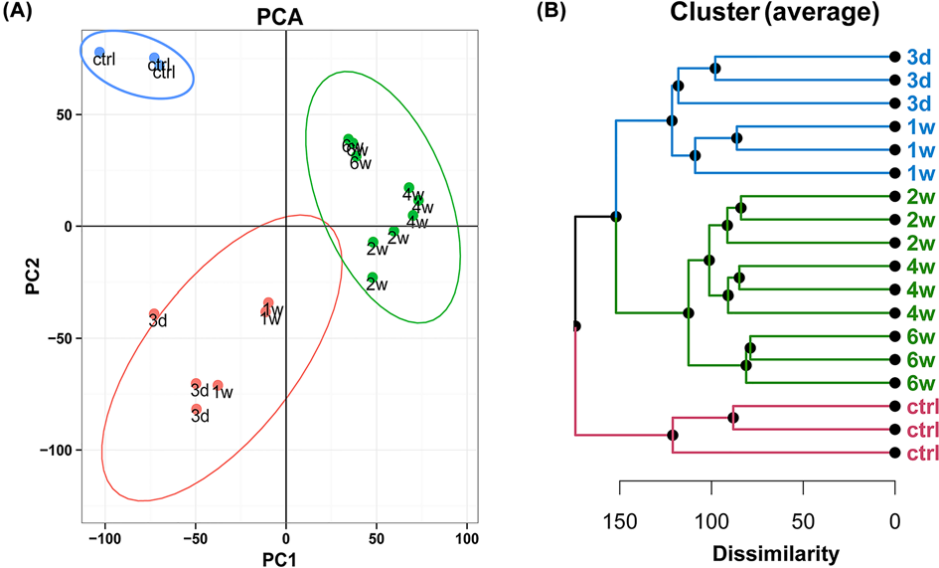
Transcriptional profiles of tendon at six observed time points (**A**) Principal component analysis (PCA) of the 20,340 probes at the six observed time points of tendon healing. (**B**) Dendrogram of gene expression profiles created with unsupervised hierarchical clustering analysis.

After filtering out the probes with low expressions, 20,340 probes were included for analysis. PCA and unsupervised hierarchical clustering analysis of all biological and experimental replicates showed that the expression profiles of the tendons at the six observed time points could be segregated into three transcriptionally distinct clusters. The first cluster included the control tendons without surgery, the second cluster included injured tendons on postoperative day 3 and week 1, and the third cluster included injured tendons at postoperative weeks 2, 4, and 6 (Figure 1A,B). The transcription profiles of the postoperative groups were all visibly distinct from the control group. Cluster analysis revealed distinct transcriptome patterns of the tendons after injury at the different stages of the healing process.

**Figure 2:**
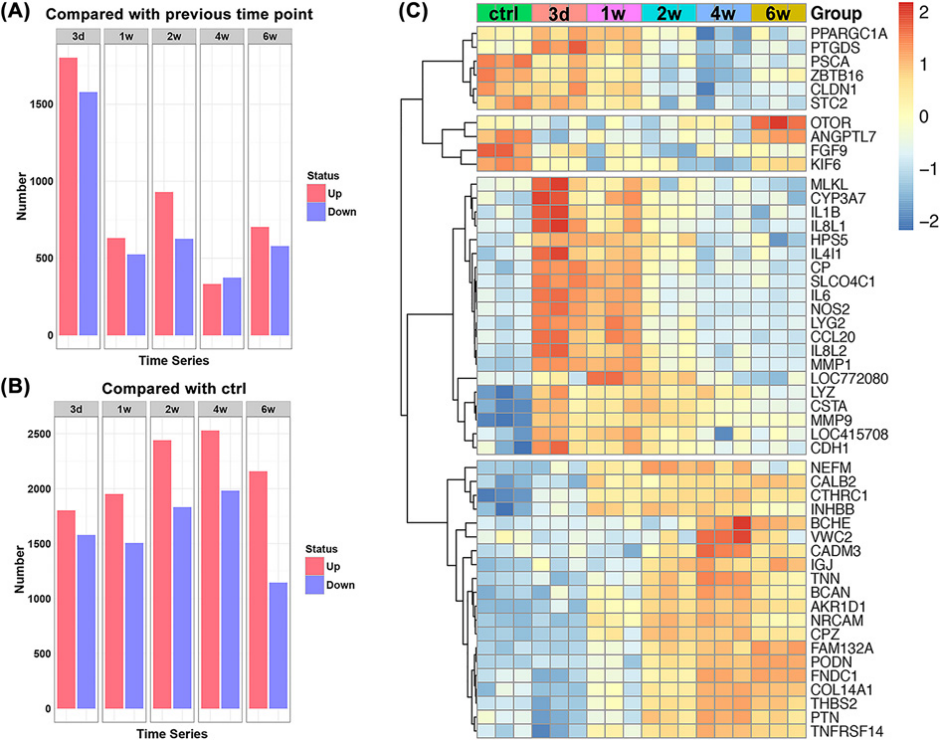
Dynamic transcriptome changes and significant differential expessed genes during tendon healing Number of differentially expressed genes with *P*<0.05 and 2-fold change at each time point relative to (**A**) its previous time point and (**B**) the control. (**C**) Heatmap of the top 50 differentially expressed genes over the entire course of tendon healing, which can be divided into four groups according to the gene expression profiles.

One-way ANOVA was conducted to gain insight into the dynamic transcriptome changes during tendon healing. A total of 6895 probes exhibiting significant changes over the course of tendon healing were identified as differentially expressed genes (*P* value<0.05, 2-fold change). Each time point was compared with its previous time point or with the control to determine the number of differentially expressed genes (Figure 2A,B). Overall, the number of up-regulated genes was slightly greater than down-regulated genes. The largest number of differentially expressed genes compared with the control was observed on day 3, at which 1802 probes were up-regulated and 1579 were down-regulated. Most of the transcriptional changes occurred on day 3, indicating that tendon response to injury might be drastically triggered at this stage. From day 3 onwards, although the number of differentially expressed genes remained high, the number of genes was comparable to that of day 3, i.e., tendon response to injury became moderate and steady from weeks 1 to 6 after injury.

Empirical Bayes model was used to rank genes based on their expression variance over time. Heat map for the top 50 differentially expressed genes ranked by HotellingT2 value in descending order revealed that these genes showed four different expression patterns (Figure 2C). Six genes were highly expressed within 1 week after tendon injury and were mainly involved in energy metabolism (*PPAEGC1A and STC2*) and cell cycle progression (*PSCA and ZBTB16*). Four genes were expressed at low levels from day 3 to week 4, of which 2 genes were known to encode proteins crucial for angiogenesis and mitogenesis (*ANGPTL7 and FGF9*). Intriguingly, *OTOR* expression remained low until week 6 at which the level increased notably. Since *OTOR* is involved in cartilage development and maintenance, its potential role in tendon regeneration would be an interesting topic in future studies. The expression of 20 genes increased markedly between day 3 and week 1, and a substantial portion of these genes were associated with inflammatory process (*IL1B, IL8L1, IL4I1, IL6, NOS2, CCL20, IL8L2, and LYZ*). Unexpectedly, three genes (*HPS5, CSTA, and CDH1*) closely associated with cell–cell or cell–ECM adhesions were activated as early as on day 3. Although adhesions usually form during the middle and late stages of tendon healing, the activation of these genes early in the healing period may be crucial for subsequent adhesion formation. Lastly, the expression of 20 genes remained at low levels until week 2 and increased thereafter. Most of these genes were closely related to ECM activities (*NEFM, TNN, BCAN, and COL14A1*) and cell adhesion (*VWC2, CADM3, NRCAM, and THBS2*).

### Functional and pathway enrichment of differentially expressed genes

**Figure 3:**
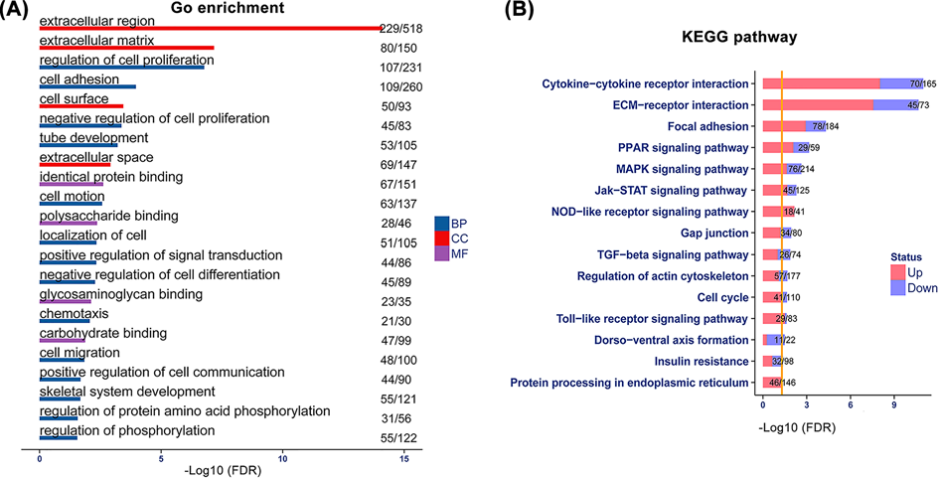
Functional annotations of differentially expressed genes during tendon healing (**A**) Gene ontology (GO) terms enrichment; BP, biological processes; CC, cellular component; MF, molecular function. Number/number: the number of differentially expressed genes/the total number of genes contained in the GO category. (**B**) Kyoto encyclopedia of genes and genomes (KEGG) pathway enrichment. Yellow vertical line displays the position when *P*=0.05. Up: up-regulated, Down: down-regulated. Number/number: the number of differentially expressed genes/the total number of genes in the pathway.

To associate the changes of gene expressions with biological functions, we conducted a GO enrichment analysis to determine the enriched GO terms. Differentially expressed genes were categorized by their functions into biological processes, cellular component, and molecular function (Figure 3A). GO categories with a false discovery rate (FDR)<0.05 were selected. We found that the top 3 categories of biological processes were related to regulation of cell proliferation and cell adhesion, suggesting the importance of these events during tendon healing. For cellular components, all the categories were related to ECM, including extracellular region, ECM, cell surface, and extracellular space. For molecular function, the majority of GO categories were annotated as protein or carbohydrate binding.

Further analysis revealed that the differentially expressed genes were involved in different signaling pathways, and 15 key KEGG pathways were enriched with a *P* value<0.05 (Figure 3B). The most impacted pathways were cytokine–cytokine receptor interaction, ECM–receptor interaction, and focal adhesion. Most of these pathways were significantly up-regulated rather than down-regulated, which indicated their active involvement in various activities after tendon injury. For example, cytokine–cytokine receptor interaction participates in inflammation, host defense, cell growth, cell death, cell differentiation, angiogenesis, and repair processes, with the ultimate function of restoring homeostasis. ECM–receptor interaction pathway directly or indirectly controls cellular activities, including adhesion, migration, differentiation, proliferation, and apoptosis. Focal adhesion pathway plays essential roles in cell motility, proliferation, differentiation, and survival, and the signaling events involved in these processes culminate in reorganization of the actin cytoskeleton. Interestingly, some changes in cell morphology and motility, as well as modulation of gene expression, in the focal adhesion pathway are initiated by the binding of growth factors to their respective receptors, indicating the considerable cross-talk between adhesion- and growth factor–mediated signaling.

### Module analysis based on WGCNA

**Figure 4:**
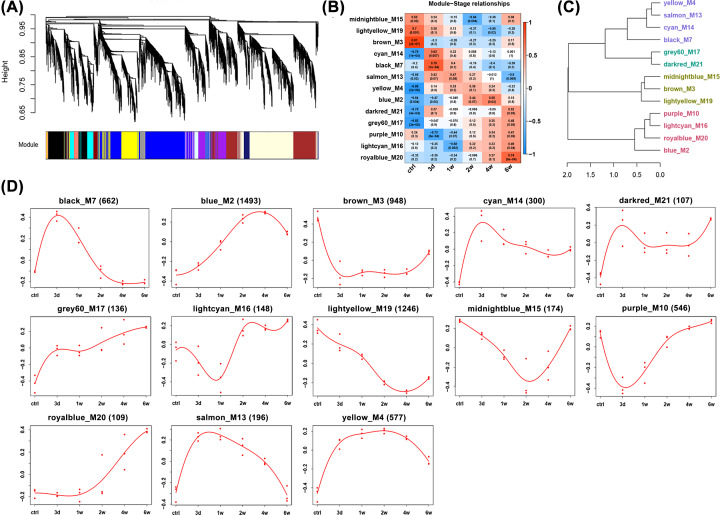
Coexpression network analysis of molecular profile changes after tendon injury (**A**) Hierarchical cluster tree showing distinct modules of co-expressed genes. Modules correspond to branches and are labeled by colors underneath the tree. (**B**) Correlation matrix of the module eigengene (ME) and time point after tendon injury. Columns correspond to modules and rows correspond to time points (red denotes positive correlation; blue denotes negative correlation). Within each cell, upper value represents the correlation coefficient; lower value represents the corresponding *P* value. (**C**) Hierarchical cluster of 13 modules. All the modules are clustered into four classes showing in different colors. (**D**) Expression trajectory of 13 modules based on ME (first principal component of gene expression).

After identifying the transcriptomic changes of individual genes, we next performed WGCNA to determine the co-expression relationships between differentially expressed genes from a system perspective. This unsupervised and unbiased analysis could reveal distinct modules of highly co-expressed genes that shared similar biological functions and facilitate the identification of hub genes that were of fundamental importance to module function. A total of 13 distinct gene co-expression modules containing 107 to 1493 genes per module were identified (labeled by color, Figure 4A,C,D; Supplementary Table S1). Module–stage association analyses revealed that 9 out of 13 modules (midnightblue, salmon, blue, and lightcyan were excluded) showed stage-specific expression values, indicating that these modules comprised genes that tended to be overexpressed or underexpressed in a single stage (|*r*|>0.7 and *P*<1e-3, Figure 4B). Genes in these nine stage-specific modules might be part of the core gene networks in each stage. Supplementary Table S2 shows the functional annotations of each module. A total of 177 genes with kME≥0.95 and *P*≤1e-8 were considered as hub genes across modules because they were highly connected and centrally located in the respective modules (Supplementary Table S3).

**Figure 5:**
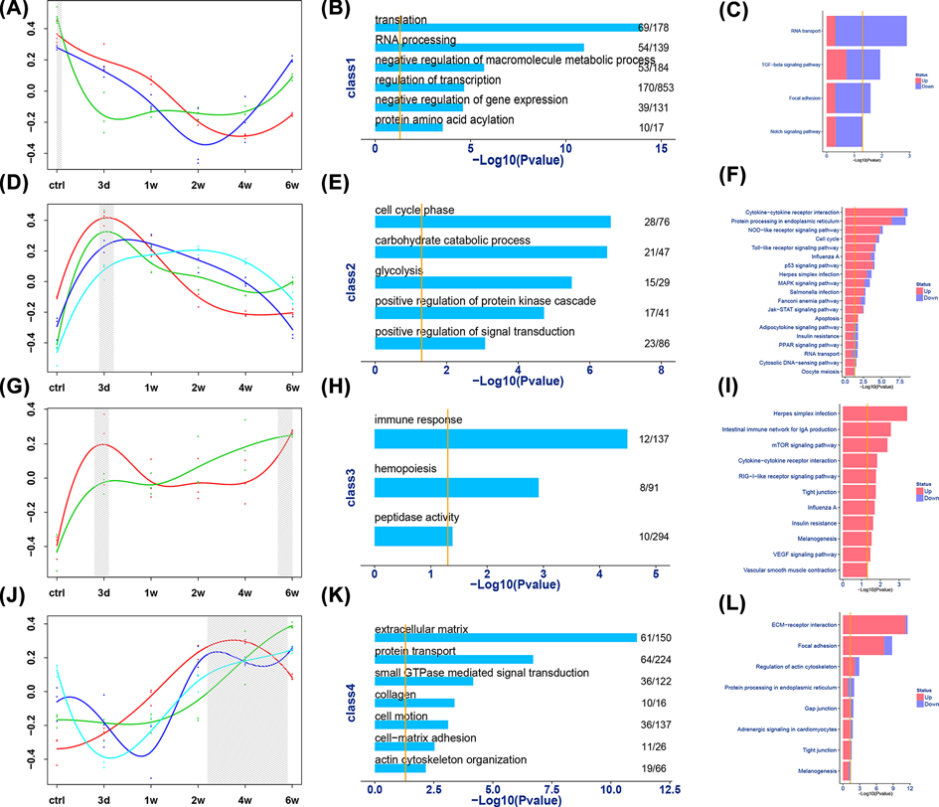
Four classes clustered according to expression trajectory and functional annotations of each class Expression trajectory of class 1 (**A**), 2 (**D**), 3 (**G**), and 4 (**J**). Grey columns show the gene expression peak in each class and represent the corresponding stage of each class. GO analysis of class 1 (**B**), 2 (**E**), 3 (**H**), and 4 (**K**) and KEGG pathway analysis of class 1 (**C**), 2 (**F**), 3 (**I**), and 4 (**L**). Yellow vertical line denotes *P*=0.05.

As some modules with similar temporal expression patterns shared certain common biological functions, we further grouped all the 13 modules into 4 classes and functionally categorized each class (Figures 4C and 5). Brown, light yellow, and midnight blue modules in class 1 contained genes undergoing gradual degradation until week 4 and were enriched for various GO categories, including translation, RNA processing, negative regulation of macromolecule metabolic process. This class was significantly enriched in KEGG pathways related to RNA transport, transforming growth factor-β (TGF-β) signaling pathway, focal adhesion, and Notch signaling pathway. In contrast, black, cyan, yellow, and salmon modules in class 2 had anticorrelated trajectories and comprised genes with the highest expression on day 3. This set of genes was significantly enriched in GO categories associated with cell cycle phase, carbohydrate catabolic process, and glycolysis. KEGG pathways enriched in class 2 included cytokine–cytokine receptor interaction, protein processing in endoplasmic reticulum, and nucleotide-binding oligomerization domain-like (NOD-like) receptor signaling pathway. Darkred and grey60 modules in class 3 contained genes that had two expression peaks during tendon healing—one on day 3 and the other at week 6 after injury. This class of genes was enriched for GO categories of immune response, hemopoiesis, and peptidase activity, and for KEGG pathways of cytokine–cytokine receptor interaction, mammalian target of rapamycin (mTOR) signaling pathway, and vascular endothelial growth factor (VEGF) signaling pathway. Blue, purple, royal blue, and light cyan modules in class 4 encompassed genes whose expression were initially low but increased gradually between weeks 1 and 6. GO categories in this class were mainly associated with matrix, including ECM, protein transport, and small GTPase–mediated signal transduction. This class was enriched with several important KEGG pathways, including ECM–receptor interaction, focal adhesion, and regulation of actin cytoskeleton.

### PPI network construction

**Figure 6:**
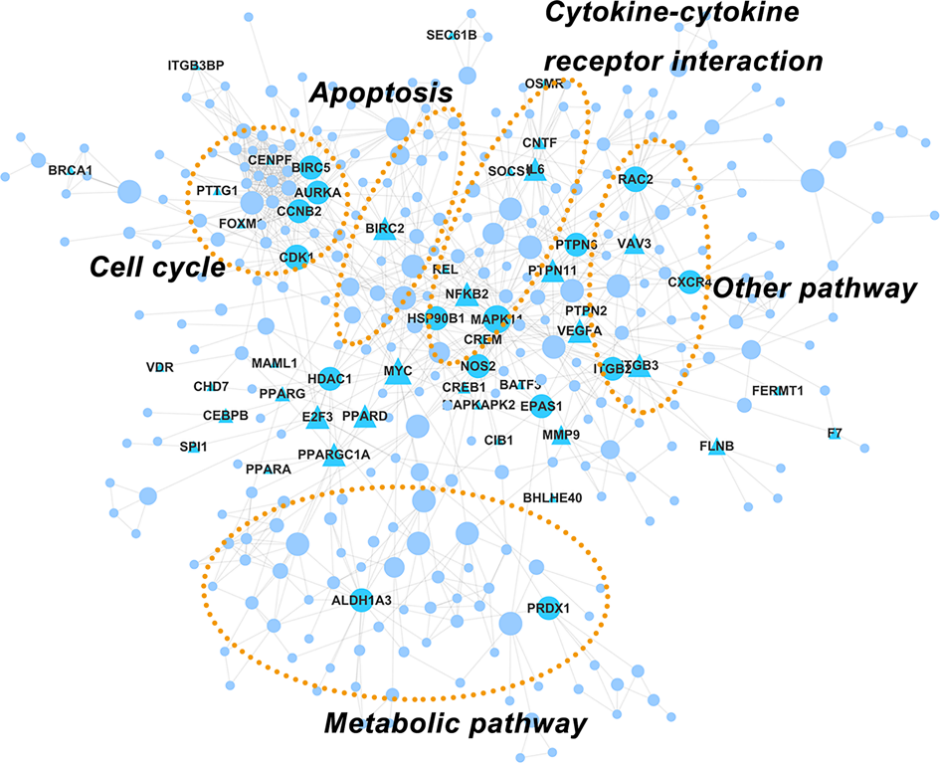
The protein–protein interaction (PPI) network of differentially expressed genes in class 2 Nodes correspond to genes and edges to PPI. Triangular nodes represent transcription factors. Larger node represents higher betweenness centrality.

To gain further insight into the datasets of classes 2 and 4 and to obtain a greater understanding of how genes in each class and stage relate to each other throughout tendon healing, we constructed PPI networks to identify specific proteins and the potential protein signaling pathways involved. For class 2 modules, a PPI network consisting of 349 nodes and 966 edges was obtained (Figure 6), of which a higher connectivity was observed when compared with a random PPI network consisting of a similar number of nodes (clustering coefficient was 0.383 and 0.017, respectively; graph density was 0.013 and 0.007, respectively). We also noted the enrichment of several important signaling pathways that contributed to the regulation of tendon repair, including cell cycle, apoptosis, cytokine–cytokine receptor interaction, and metabolic pathways. As proteins with high betweenness centrality were believed to play important roles in the network, 48 of such proteins were selected (Supplementary Table S4), among which *MAPK11, MYC, RAC2, CDK1, NFKB2*, and *VEGFA* were shown to be of high importance in the network. Furthermore, *CDK1* and *RAC2* had high kME values (>0.95), indicating that they were also ‘hub’ genes in the co-expression network.

**Figure 7:**
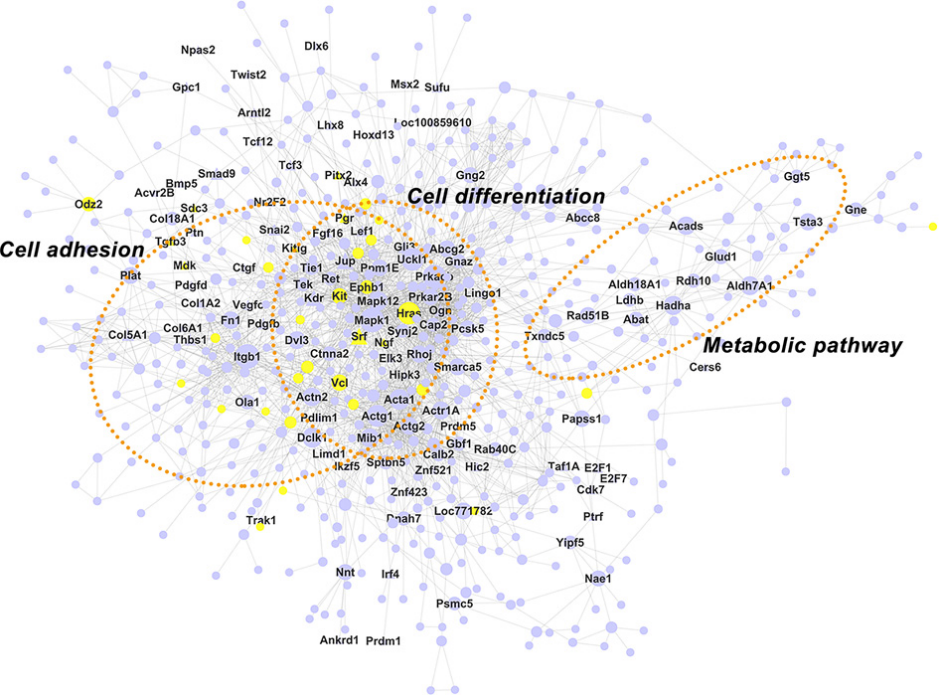
The PPI network of differentially expressed genes in class 4 Nodes correspond to genes and edges to PPI. Triangular nodes represent transcription factors. Larger node represents higher betweenness centrality.

For class 4 modules, a PPI network with 598 nodes and 2402 edges was obtained (Figure 7), which also displayed a higher connectivity compared with a random network (clustering coefficient was 0.21 and 0.014, respectively; graph density was 0.012 and 0.0067, respectively). Functional annotation analysis found that most of the proteins were related to cell adhesion, cell differentiation, and metabolic pathway, among which cell adhesion accounted for most of the key proteins. Interestingly, some proteins, such *EPHB1, PGR*, and *KITLG*, were related to both cell adhesion and cell differentiation. A total of 129 proteins were considered pivotal in the network (Supplementary Table S4) and 53 proteins were closely related to other proteins with a degree of interaction ≥10. *ITGB1, MAPK1, PRKACB, HRAS, ACTG1*, and *ACTA1* were the most important proteins in the network based on their high values of betweenness. These genes were all directly or indirectly related to cell adhesion.

### Enrichment of specific functions based on annotated modules

**Figure 8:**
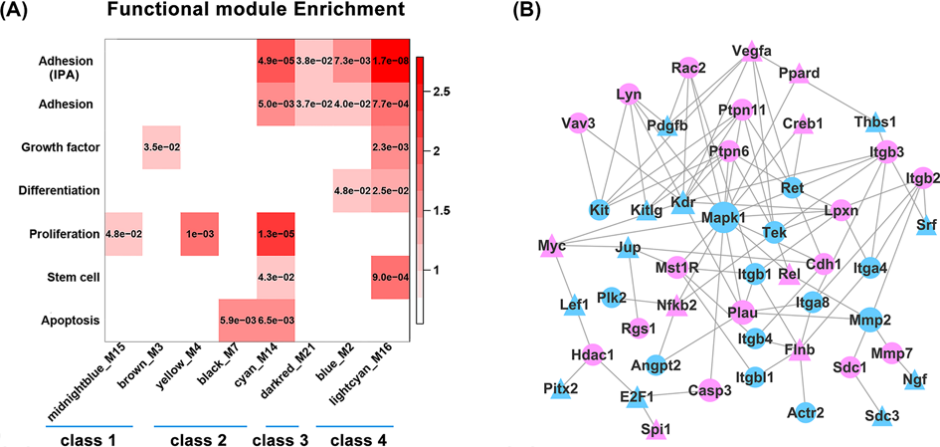
Enrichment of modules for selected biological process and PPI network of adhesion-related genes in class 2 and 4 (**A**) Module-level enrichment for specific biological process related gene sets curated from IPA or GO database (*P*<0.05). Filled colors from red to white represent *P* value from low to high. (**B**) Construction of PPI network of adhesion-related genes using STRING database.

Our previous studies have focused on several biological activities during tendon healing, including cellular proliferation and apoptosis, changes in major growth factors, and adhesion formation after tendon injury. In the past 10 years, many reports have demonstrated that tendon stem cells are multipotent and may become promising seeding cells for tendon tissue. Therefore, we selected certain biological functions of interest to study their enrichments in all the modules (Figure 8A). Genes belonging to the selected biological functions were collected from GO annotations. As adhesion was a focus in tendon healing, we simultaneously collected adhesion-related genes from the Ingenuity Pathways Analysis (IPA) database to obtain the enrichment profiles. Figure 8A shows that these functional categories distribute unevenly in the 8 modules. In terms of individual module, more functional-related genes enriched in the cyan and light cyan modules belonged to classes 2 and 4, respectively. Functionwise, the proliferation and adhesion categories were enriched in more modules with relatively higher significance compared with other categories.

Due to our special interest in adhesion formation, we analyzed the adhesion-related genes with both the GO and IPA databases. The analyses showed that these genes were similarly enriched in these 2 databases. The adhesion category ranked first in the light cyan module that belonged to class 4 and second in the cyan module that belonged to class 2. As the expression trends of the genes in classes 2 and 4 were entirely different, we were interested to find out if the adhesion-related genes overexpressed transiently on day 3 could be related to or potentially regulate the genes that overexpressed after week 2. Therefore, we selected the genes belonging to ‘adhesion (IPA)’ in classes 2 and 4 and constructed the PPI networks of these genes using the STRING database. Considering the importance of upstream regulators, only the upstream regulators and their target genes were included in the network (Figure 8B). The results showed that upstream regulators, such as *MYC, NFKB2, VEGFA*, and *FLNB* in class 2 were closely associated with the adhesion-related genes in class 4. Conversely, *MAPK1, ITGB1*, and *MMP2* in class 4 were closely related to the adhesion-related genes in class 2.

### Validation by quantitative real-time PCR

**Figure 9:**
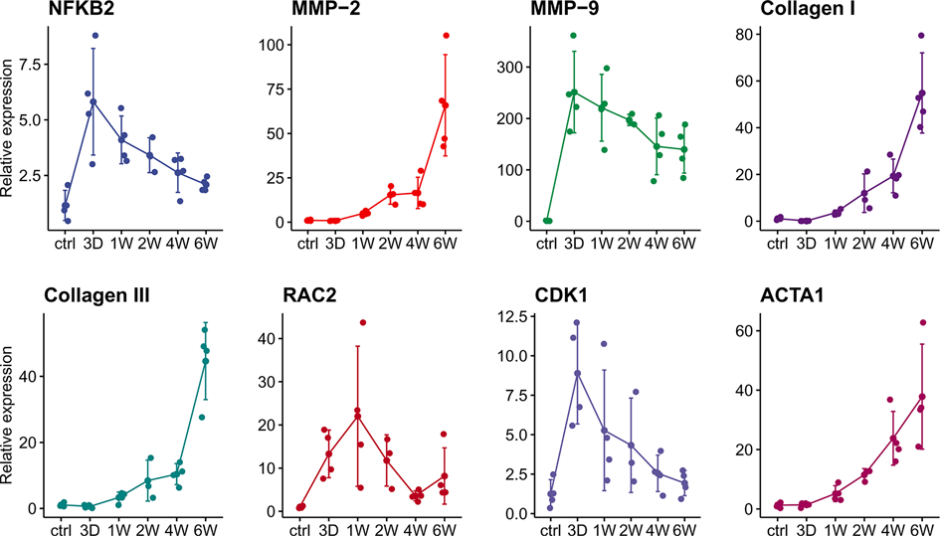
Gene expression level of NFKB2, MMP2, MMP9, collagen I, collagen III, rac2, cdk1, and acta1 by qPCR Results of real-time PCR analyses clearly corresponded to those of the Affymetrix analyses.

We verified the Affymetrix data by examining the expression levels of the representative genes enriched in class 2 and 4 (NFKB2, MMP2, MMP9, collagen I, collagen III, rac2, cdk1, and acta1) using the quantitative real-time PCR (qPCR) method. After comparison, results obtained from the real-time PCR analyses clearly corresponded to the results of the Affymetrix analyses (Figure 9).

## Discussion

The present study was designed to provide a system-level perspective of gene expression changes during the tendon repair process. Tendon healing consists of a series of complicated cellular and molecular events driven by a tremendous number of genes. Previous studies have proposed three successive phases after tendon injury—inflammatory, proliferative, and remodeling phases [25,26]. Inflammatory phase starts immediately after injury and lasts for about a week. Proliferative phase starts from 5 days after injury and lasts for about 6 weeks. Remodeling phase starts from around 4 weeks and lasts for several months or longer. This theory is mainly derived from the observed morphological changes of the tendons and the detection of specific biomolecular markers during tendon healing. The transcriptional landscape of the tendons after injury is largely unknown. In the present study, we aim to provide a big picture on gene expression changes during a 6-week period of tendon healing, which will expand our current understanding of the molecular mechanisms involved in tendon repair.

We first identified 6895 genes that were differentially expressed during the course of tendon healing. PCA and hierarchical clustering analysis clearly segregated the observed healing period into three transcriptionally distinct stages. The first stage corresponded to the normal control group, the second stage corresponded to day 3 to week 1 after injury, and the third stage corresponded to weeks 2–6 after injury. These three stages were aligned with the three phases of tendon healing described in previous reports. The results of the present study represent our interpretation based on the overall understanding of the tendon repair process at the molecular level. Further investigations on the relationships and transitioning of these three transcriptional phases may provide useful information on how to modulate the accessibility of crucial transcription machinery.

Comparisons of gene expression between two adjacent time points, or between each postoperative time point and the control, showed that the numbers of differentially expressed genes remained high during the tendon healing period. The largest change in gene number was observed on day 3, indicating that most reactions occurred within the first 3 days after tendon injury. It is believed that inflammatory response plays a predominant role during the early period of tendon healing. Therefore, we speculated that the genes with significant changes in expression on day 3 should play critical roles in inflammatory reactions. Between weeks 1 and 6, the number of differentially expressed genes maintained at similar levels. During this period, the injured tendons undergo a slow but gradual regenerative process, which has been reported to involve cell proliferation, collagen deposition, and ECM remodeling [27,28].

The top 50 differentially expressed genes represented the most active genes participating in the tendon repair process. These genes displayed four distinct expression trends, which largely corresponded to the three transcriptionally distinct stages determined above. For instance, the 20 genes with extremely high expression on day 3 and week 1 after tendon injury are mainly involved in inflammatory response, which is the major event during the early inflammatory phase of tendon healing. Furthermore, another 20 genes with high expression after week 2 are closely related to ECM activities and cell adhesion, which are the main events during the regenerating and remodeling phases of tendon healing. Therefore, these functionally diverse genes not only constitute an important inventory of tendon markers that can characterize the distinct phases of tendon healing, but also provide us with new directions to further the research of tendon repair.

Go analysis and KEGG databases are dedicated to study the function of large-scale transcriptomic or genomic data [29,30]. We found that cell proliferation and cell adhesion were the most enriched biological processes after tendon injury. These findings are consistent with the previous studies showing that tissue regeneration and adhesion formation are the two main processes during tendon healing. Notably, interactions between cells and ECM are the most significant events in cellular components. KEGG pathways analysis shows that cytokine–cytokine receptor interaction is the most enriched pathway during tendon healing. Cytokines are crucial intercellular regulators that induce responses through binding to their specific receptors [31]. They are implicated in the acute and chronic inflammatory reactions after tendon injury [32,33]. ECM–receptor interaction and focal adhesion are the second most enriched pathways, which echoes the top GO categories in biological processes. Collectively, these results reveal that adhesion formation is the major event in tendon repair. In addition, we also found that the VEGF and TGF-β pathways, which had been the focus in many previous studies, were both enriched in our KEGG pathways analysis [34–37].

To better understand gene co-expressions during the tendon repair process, WGCNA was used to elucidate highly co-expressed modules and to identify hub genes in the respective modules. WGCNA not only quantifies the correlations between the expressions of individual genes, but also calculates the extent to which these genes share the same neighbors in the network [38]. We identified 13 modules and 177 hub genes across the modules. These hub genes may be critical within the network and play key roles in biological activities. To simplify the data, we decided to group the modules into four classes according to the similarities in gene expression trends and biological activities. Our results showed that these four classes could clearly delineate different stages of tendon healing based on their respective gene expression peaks: class 1 corresponded to the control, classes 2 corresponded to day 3 and week1, and class 4 corresponded to weeks 4 and 6. These results were similar to the PCA analysis. Through conducting GO and KEGG pathway enrichment analyses, we detailed the biological functions and key pathways involved in each class.

Following tendon injury, the weak self-repair capacity during the early healing period and adhesion formation during the middle and late healing periods are the two main clinical issues in tendon healing. Researchers have been focusing on modulating these two issues for years, both at the protein and gene levels [39–42]. Thus, it is more meaningful to focus the present study on the early response within 1 week after tendon injury and the late response from 4 to 6 weeks after tendon injury. Through WGCNA analysis, we found that transcriptomic changes in class 2 represented the early response after tendon injury and the changes in class 4 represented the middle and late responses of tendon healing. The expression of genes in class 2 increased immediately after tendon injury and peaked on day 3. These genes were actively involved in a variety of activities, such as cytokine–cytokine receptor interaction, cell cycle, apoptosis, and immune responses–related pathways. The expression of genes in class 4 showed a significant upward trend from weeks 2 to 6 and the biological phenomena involved were mainly associated with ECM activities and adhesion. The results of the present study are consistent with previous studies reporting the early and late responses of tendon healing. Therefore, we focused our attention on the analysis of classes 2 and 4 modules. Class 3 was intriguing because the genes in this class were highly expressed at two discontinuous time points (day 3 and week 6), indicating that some genes played multiple roles in more than one phase of tendon healing and that similar biological changes occurred between weeks 3 and 6. Functional annotations showed that class 3 genes were mainly related to immune response. As inflammatory reactions are the predominant responses during tendon healing, these results signify that inflammation is a persistent and long-term process after tendon injury. However, the number of genes in class 3 was small and functional enrichment was of little significance; therefore, this class was not further analyzed.

In view of the importance of classes 2 and 4 modules, we next constructed PPI networks of genes included in these two classes. Genes that have high centrality were highlighted in the networks. The schematic diagrams provided us with an overview of the key signaling pathways and hub genes involved in each class. The results showed that the key enriched pathways in the network of class 2 were cytokine–cytokine receptor interaction and apoptosis, and the hub genes in class 2 were *MAPK11, MYC, RAC2, CDK1, NFKB2*, and *VEGFA*. The key enriched pathways in the network of class 4 were cell differentiation and cell adhesion, and the hub genes in class 4 were *ITGB1, MAPK1, PRKACB, HRAS, ACTG1*, and *ACTAL1*. These networks highlighted the interconnectivity of genes that were differentially expressed at different stages of tendon healing and provided insight into the importance of these genes. For instance, integrins (ITGs) are transmembrane receptors that facilitate cell–ECM adhesion and have two main functions: attach the cell to the ECM and transduce signals from the ECM to the cell [43,44]. The biological functions of ITGs family are mainly associated with adhesion. In the present analysis, we found that ITGs were the key genes in class 4, which represented the critical stage of adhesion formation after tendon injury. This suggests that ITGs may serve as novel targets in future investigations on adhesion formation.

Finally, we focused on several biological activities of interest, including adhesion, growth factor, differentiation, proliferation, stem cell, and apoptosis, and studied their specific enrichment in all the modules. The results showed that most of the selected functions were enriched in classes 2 and 4, while classes 1 and 3 contained notably fewer functions of interest and with relatively lower significances. Unexpectedly, adhesion was enriched in both classes 2 and 4, which exhibited very different gene expression patterns. To clarify the relationships of the adhesion-related genes in these two classes, we constructed the PPI networks of these genes extracted from the IPA database. The results showed that *MYC, NFKB2, VEGFA*, and *FLNB* in class 2 were closely related to the adhesion-related genes in class 4, and *MAPK1, ITGB1*, and *MMP2* in class 4 were closely related to the adhesion-related genes in class 2. It would be interesting to delineate the effects of these early differentially expressed genes on adhesion formation, which only occurs during the late period of tendon healing, in our future investigations. For example, *NFKB2* encodes a subunit of the transcription factor complex nuclear factor-kappa-B, which functions as a central activator for genes involved in inflammation and immune function [45,46]. In the PPI network, *NFKB2* is not only an active gene participating in adhesion activities, but also a key upstream transcription factor modulating the functions of the downstream adhesion-related genes. This phenomenon warrants further investigations.

The present study provides a comprehensive summary of the genes activated after tendon injury in chickens. Taken together, 6895 genes were differentially expressed over a 6-week period of tendon healing. The main biological processes involved were regulation of cell proliferation and cell adhesion, and the most altered pathways were cytokine–cytokine receptor interaction and ECM–receptor interaction. Four distinct patterns of gene expression were identified through WGCNA analysis, and genes with marked increase in expression at week 6 were closely associated with cell adhesion and ECM activities. Interestingly, some adhesion-related genes, such as *MYC, NFKB2, VEGFA*, and *FLNB* were highly expressed on day 3 and were closely associated with genes in the late adhesion process. This inspired us to further our studies on adhesion formation during tendon healing. The microarray data demonstrated its powerful potential for advancing our knowledge on tendon healing and predicting regulatory targets for the genetic engineering of tendon repair. The limitation of the present study is that there were three tendon samples at each time point in the microarray assay. Although increasing the number of samples would strengthen the bioinformatic analysis, we beard in mind the need to reduce the use of animals to a necessary minimum for a microarray technology.

## Conclusions

The comprehensive analysis of the biological functions and interactions of the genes differentially expressed during tendon healing can broaden our understanding on the tendon repair process. We believe that the microarray data have demonstrated its powerful potential for predicting regulatory targets for the genetic engineering of tendon repair.

## Supplementary Material

Supplementary Tables S1-S4

## Data Availability

All data generated or analyzed during this study are included in this published article and its supplementary information files.

## Abbreviations

ECM: Extracellular Matrix
GO: Gene Ontology
IPA: Ingenuity Pathway Analysis
KEGG: Kyoto Encyclopedia of Genes and Genomes
PCA: Principal Component Analysis
PPI: Protein–protein Interaction
WGCNA: Weighted Gene Co-expression Network Analysis
